# Grape Sugar and Glucose

**Published:** 1882-06-20

**Authors:** J. M. Tallman


### GRAPE SUGAR AND GLUCOSE.

BY J. M. TALLMAN.

The following sketch is not intended by the writer to be a complete description of the above named articles of commerce which have recently become such important factors to the grocery, confectionery and brewing interests; but it will simply cover the important features of the process of manufacturing and to give to the outside world a litttle light respecting what will become, and in fact is, one of the important industries of the day.

The terms grape sugar and glucose are used to designate the two forms in which the article is sold by the manufacturer. Grape sugar is the solid and glucose the liquid form. It is made from starch by the action of sulphuric acid, or oil of vitrol. The first stage in the manufacture is the production of starch. This is made from the Indian corn of commerce ; that which possesses the largest amount of starch having the preference over corn of smaller kernels and consequently less starch.

The corn separated from the cob is placed in large vats with boiling water, and there undergoes a steeping process of about forty-eight hours duration, during which time the water is changed occasionally ; this serves to swell the kernel to nearly twice its natural size and softens it very materially.

The mass is then run through mill-stones, which grind it to a fine pulp. With a large percentage of water in this, there are the starch, gluten and woody fibre, etc. In order to separate the starch from this, the pulp is run over large frames covered with bolting cloth, similar to that used in flour mills; several small streams of water are also introduced, and the water falling on the pulp as it passes over the sieve, separates the starch from the hull which passes off, the starch falling into a trough underneath, from whence it is cenveyed to tubs of larger size, where it is allowed to settle, and the water, which has by this time accumulated large quantities of dirt, etc., is allowed to drain off. Caustic soda is then added in about the proportion of 600 pounds to each bushel of corn; this serves to further separate the starch from the gluten. From the settling tubs it is allowed to run through long inclined troughs of a very slight elevation. While the water, containing large quantities of starch, gluten, etc., is passing over these tables, as they are called, the starch slowly settles to the bottom, allowing the dirt, gluten and alkali to pass away.

The now solid mass of starch is taken from the tables and after being broken up thoroughly in water, is pumped into tubs and agitated thoroughly; this further cleans it. The result of this last manipulation is a clean starch, such as is used on our tables in pudding, etc., and in our laundries to stiffen and improve our shirts and other articles. The starch mixed with water is then poured into a large tub called a *converter*, and it is here that the starch loses its identity, and in a high temperature is converted by the action of sulphuric acid into either grape sugar or glucose.

If glucose is desired the liquid is boiled until, by the application of the iodine test (known to chemists as the method for determining the existence of starch), it shows that it has very nearly lost its character as starch, and the conversion is then stopped by drawing off the liquid. Should grape sugar be desired, the process is carried still further until not a trace of starch is visible, and the liquid is all converted into sugar. We will now follow it from the converting tub to another large tank in which it lies perfectly passive, but possessing in its muddy looking mass a powerful agent and the one source from which are derived the many ideas regarding its poisonous character. I refer to the sulphuric acid. Were the substance left as it now is, and the article put upon the market,it would certainly serve to be severely handled by physicians parents, and all interested in the health of the people at large. It is a well-known fact that if marble dust is introduced into a liquid containing sulphuric acid, a chemical change takes place, and if a sufficient quantity is introduced, the acid in its entirety is absorbed, and the two together become sulphate of lime, a precipitaie easily removed. This is the course pursued in neutralizing the acid, and to this end a test paper is applied to determine when the neutralization has taken place; when this is accomplished the liquid is a sweet water, quite dirty however ; it is then passed through filter cloths of various degrees of fineness, and they climinate the portion of the dirt, including the sulphate of lime. This water is then allowed to run through a larger tank containing burnt bone or charcoal. This takes unto itself what dirt has escaped the former filtering medium, and the now clear liquid although nearly as thin as water, is quite sweet. It is then introduced into a copper pan of large capacity, called a vacuum pan, and the process of evaporation goes on. As the liquid is slowly evaporated under the influence of heat, it assumes a thick, gummy character of a density of thirty-eight to forty-two degrees Beaume. It is now ready for cooling and shipping. Should it be grape sugar it is drawn into barrels or boxes, where it rapidly cools and becomes solid and perfectly white; should it be glucose, and intended for confectioners' use, it is drawn into barrels, from which, owing to its density, it is quite difficult to abstract it unless by heating. Should the manufacturer wish to fill an order calling for syrup, he mixes with the still warm glucose about ten per cent. of its bulk of cane syrup, a liquid obtained from cane sugar during the refining process. This syrup serves to sweeten the not too sweet glucose, and imparts to it a bright yellowish color called by grocerymen "golden," and so labeled on their syrup cask. The grape sugar maker sells to outside parties the sugar in solid form. They, by the use of patented machinery, cut it into minute pieces and mix it with the coffee sugars of commerce, in the proportion of eighty per cent. cane sugar and twenty per cent. grape sugar. This proportion will vary somewhat, but is in the main correct. The appearance of the mixture is a very decided improvement over the appearaoce of the pure cane sugur. due to the fact that the cane sugar, being of a yellowish color, is made to look much lighter, when mixed with the white grape. The consumer, unless very well posted, cannot detect the difference, and obtains sugar of much lighter color for the same amount of money than he would otherwise pay for a dark, yellow grade.

The confectionery trade uses a large quantity of both glucose and sugar in the manufacture of all grades of confectionery. Brewers are very large consumers, as well as jelly and wine manufacturers; printers mix glucose with glue and use it for inking their type. Thus it is rapidly becoming a popular necessity. It cheapens the food and when properly made does not hurt the stomach. There is only the one chance for it to be injurious to the human system, and that is in the existence of acid, which cannot occur if the manufacturer is careful to thoroughly neutralize it; if this is done it contains nothing that is harmful. It is estimated that the entire consumption of corn this ensuing year, for this purpose, will be between ten and eleven millions of bushels, of which quantity Buffalo alone requires about six millions.—*Phy. and Surg. Investigator.*

